# Long-term myopia control effect and safety in children wearing DIMS spectacle lenses for 6 years

**DOI:** 10.1038/s41598-023-32700-7

**Published:** 2023-04-04

**Authors:** Carly Siu Yin Lam, Wing Chun Tang, Han Yu Zhang, Paul H. Lee, Dennis Yan Yin Tse, Hua Qi, Natalia Vlasak, Chi Ho To

**Affiliations:** 1 Centre for Myopia Research, School of Optometry, The Hong Kong Polytechnic University, Hung Hom, Kowloon, Hong Kong; 2 Centre for Eye and Vision Research, Sha Tin, Hong Kong; 3 Southampton Clinical Trials Unit, University of Southampton, Southampton, UK; 4 Technical Research and Development Department, Hoya Vision Care, Tokyo, Japan; 5 Technical Research and Development Department, Hoya Vision Care, Amsterdam, The Netherlands; 6 School of Medicine, Nankai University, Tianjin, China

**Keywords:** Health care, Medical research

## Abstract

This study evaluated the long-term myopia control effect and safety in children wearing Defocus Incorporated Multiple Segments (DIMS) spectacle lenses. Participants who completed the 2-year RCT were followed for a total of 6 years; their cycloplegic refractions and axial length were measured. Group 1 (n = 36) wore DIMS spectacles for 6 years; Group 2 (n = 14) wore DIMS lens for the first 3.5 years and SV spectacles afterwards; Group 3 (n = 22) wore SV spectacles in the first 2 years and switched to DIMS; Group 4 (n = 18) wore SV spectacles in the first 2 years, switched to DIMS for 1.5 years and then SV spectacles again. Group 1 showed no significant differences in myopia progression (− 0.52 ± 0.66 vs. − 0.40 ± 0.72D) and axial elongation (0.32 ± 0.26 vs. 0.28 ± 0.28 mm, both *p* > 0.05) between the first and the later 3 years. In the last 2.5 years, DIMS lens groups (Groups 1 and 3) had less myopia progression and axial elongation than the single vision groups (Groups 2 and 4). There was no evidence of rebound after stopping the treatment. Post-wear visual functions in all groups were within norms. The results supported that DIMS lenses provided sustained myopia control without adverse effects over the 6-year study period.

Trial registration: clinicaltrials.gov; NCT02206217.

## Introduction

Myopia is now an alarming concern worldwide as it is estimated to impact more than half of the global population by 2050^1^. The prevalence of high myopia (− 5.00D or greater^2,3^) is expected to increase from 3% at present to 10% of the myopic population by 2050.^1^ High myopia is associated with an increased risk of vision-threatening pathologies^2^, such as myopic macular degeneration, which is one of the leading causes of low vision and blindness in different countries, such as European regions and China^4,5^. Thus the high prevalence of myopia brings significant public health and socio-economic problems^6,7^.

Different strategies have been suggested to delay the onset of myopia and slow myopia progression in children. Atropine is one of the popular drugs used for controlling childhood myopia progression and has shown the most efficacy among different remedies^8^. Recent clinical trials have indicated that low-concentration (0.01%) atropine eyedrops have also obtained modest treatment effects with low myopic rebound and minimal side effects^9–11^. For optical interventions, orthokeratology^12–14^, some spectacle lenses^15,16^ and soft contact lenses^17–19^ are specially designed to impose myopic defocus on the retina and have shown promising reductions in the progression rate of myopia and eye growth.

The Defocus Incorporated Multiple Segments (DIMS) spectacle lens is designed to control myopia by imposing myopic defocus with the principle of simultaneous vision. It is a dual-focus spectacle lens consisting of a central optical zone for correcting distance refractive error, and a batch of small circular segments of + 3.50D equally distributed throughout the mid-peripheral area in a honeycomb pattern^15^. Thus, the DIMS spectacle lens introduces myopic defocus and provides a clear vision for the wearer simultaneously at all distances. The 2-year double-masked randomized controlled trial (RCT) found that DIMS spectacle lens wear could slow childhood myopia progression significantly by 0.44D and axial elongation by 0.34 mm compared with regular single vision (SV) spectacle lenses wear over the evaluation period^15^. In the third year, the children in the treatment group continued to wear DIMS spectacles (DIMS group), and the results showed the slowing effect on myopia progression was sustained over 3 years^20^. On the other hand, the children in the control group switched to DIMS lens wear due to ethical concerns (Control-to-DIMS group). Their myopia progression and axial elongation in the 3rd year were significantly decreased compared with those in the first and second years. Thus, a good myopia control effect was shown in the children when they changed from SV to DIMS spectacle lens wear. However, the long-term treatment effect and safety with DIMS spectacles were uncertain.

This study aimed to monitor the refractive error and axial length (AL) as well as the safety of the children who wore DIMS spectacle lenses for 6 years and to determine if wearing the DIMS spectacle lens slows myopia progression and axial elongation throughout this period. This study also determines the effect of stopping DIMS spectacle lens wear and the changes in refractive error and axial growth in those children who reverted to SV spectacle lenses. We also evaluate any rebound effects after discontinuation of DIMS spectacle lens wear.

## Materials and methods

### Participants and study design

Hong Kong ethnic Chinese children who completed both the 2-year RCT^15^ and the 3rd year study of DIMS spectacle lenses^20^ were invited to participate in this follow-up study. Comprehensive eye examination and related ocular data collection were performed over 6 years after the initial RCT commenced. The participants were asked what types of optical lenses and myopia interventions they had. The children who changed to other myopia control methods or had any ocular anomalies were excluded from this study.

There was the intention of continual follow-up visits at 6-month intervals after the 3rd year; however, the university campus was closed due to unexpected societal events and the COVID pandemic also hit, and all the children were released from the study at 3.5 years. Children and parents were advised that they could opt for their choices of spectacle lens wear. Any follow-up activities and data collection could not be performed until May 2020. The children were invited back for the sixth-year follow-up, regardless of their current choice of spectacle lens wear (DIMS or SV spectacles). Data collection was completed in October 2021.

Participants were divided into 4 groups (eTable 1). Group 1 wore DIMS spectacles from 0 to 6 years; Group 2 wore DIMS spectacles from 0 to 3.5 years and changed to wearing SV spectacles afterwards; Group 3 wore SV spectacles in the first 2 years and switched to DIMS spectacles afterwards; Group 4 wore SV spectacles in the first 2 years, switched to wear DIMS spectacles for 1.5 years and then switched to SV spectacles again. Changes in spherical equivalent refraction (SER) and AL over 6 years were analyzed and compared.

### Study procedures and data collection

As for the previous RCT of DIMS spectacle lens, all data collection was carried out at the Centre for Myopia Research, The Hong Kong Polytechnic University. All procedures of the study followed the tenets of the Declaration of Helsinki. This study was approved by the Human Subjects Ethics Sub-committee of The Hong Kong Polytechnic University (HSEARS20191008002) before the commencement of the study. Written assent and informed consent were obtained from the children and their parents after explanations of the nature and possible consequences of the study.

The primary and secondary outcomes were SER (D) and AL (mm) changes. Data collection procedures followed those in the previous trials of DIMS lenses. SER was measured by cycloplegic auto-refraction using an open-field autorefractor (Shin-Nippon NVision-K5001, Ajinomoto Trading Inc.), whereas AL was measured by partial coherence interferometry using an IOL Master (Carl Zeiss Meditec). One drop of Alcaine 0.5% and then 1–2 drops of cyclopentolate HCL 1% were instilled to induce cycloplegia. An average of five autorefraction and AL measurements for each eye were used for data analysis.

Other measurements such as distance and near visual acuities (VA), distance and near phoria, stereoacuity and amplitude of accommodation (AA) were performed when the children were wearing full distance correction based on non-cycloplegic subjective refractions.

### Statistical analysis

SPSS statistical analysis software, version 26 (IBM Corp., Armonk, NY, USA), was used for data analysis. The means and standard deviations (SD) of all continuous variables are presented unless otherwise stated. Only data from the right eyes were presented. Myopia progression over 6 years in each group was calculated as the difference between SER at 6 years and baseline. The cumulative myopia progression and axial elongation per 6 months over 6 years were calculated. The trend of changes in SER and AL was plotted against time. The changes in SER or AL between 3.5 and 6 years were the differences between SER or AL at the 6-year visit and the 3.5-year visit. The changes in SER and AL were compared among 4 groups.

## Results

### Participant number and demographic data

**Figure 1:**
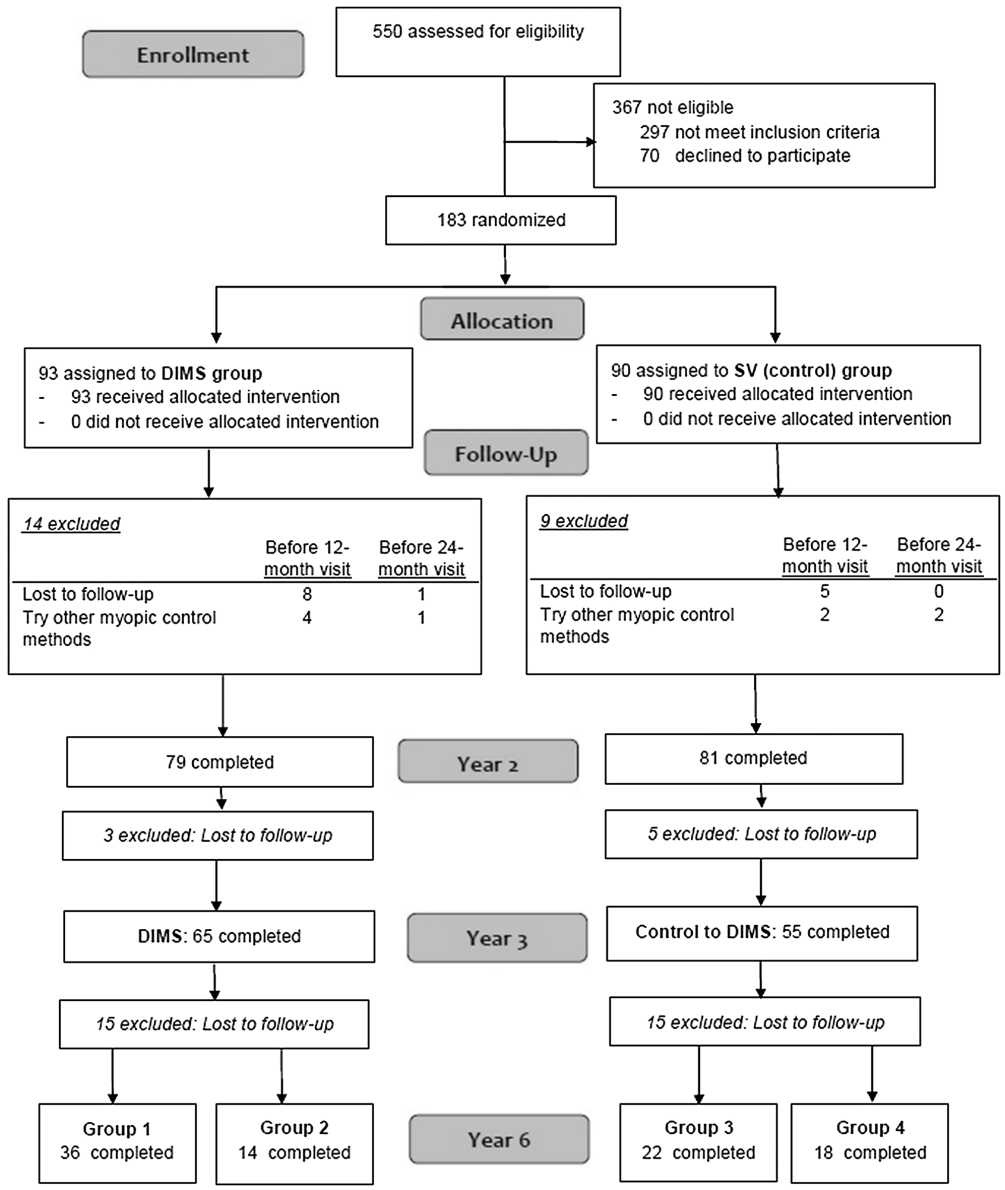
Flowchart showing treatment allocation and participant number in different stages of the DIMS study over 6 years. Both DIMS and Control-to-DIMS groups wore DIMS lenses in year 3. Group 1: wore DIMS lenses for 6 years, Group 2: wore DIMS lens for the first 3.5 years and SV spectacles afterwards. Group 3: wore SV spectacles in the first 2 years and switched to DIMS, Group 4: wore SV spectacles in the first 2 years and switched to DIMS for 1.5 years and then SV spectacles again.

Figure 1 shows the number of participants and the loss to follow-up. 120 children who completed the 3rd year trial (DIMS, n = 65; Control-to-DIMS, n = 55) were invited to join this follow-up study. A total of 92 children (77%) enrolled, and 28 (23%) did not join or were excluded (eTable 2). Most of the children (n = 20) were not willing or too busy to come back for an eye examination; 3 children studied abroad and 4 children changed to other methods of myopia control (3 changed to orthokeratology and 1 child used atropine eye drops). Additionally, 1 child in the DIMS group was excluded due to suffering from ocular disease. Finally, 90 children completed the 6-year data collection.

Table 1 summarizes the demographic findings, mean SER and AL at different visits. No statistically significant differences were found in age, gender, SER and AL at baseline and year 3 (*p* > 0.05) between groups. All children wore their spectacles daily full-time (at least 15 h/day on average).

**Table 1:** Summary of demographic data for the dropouts and the children who completed the 6-year follow-up study.

Mean ± SD	Dropouts (N = 15)	DIMS		Dropouts (N = 15)	Control-to-DIMS		*P*
		Group 1 (N = 36)	Group 2 (N = 14)		Group 3 (N = 22)	Group 4 (N = 18)	
Age at enrolment, years	10.80 ± 1.57	9.75 ± 1.42	10.21 ± 1.53	9.73 ± 1.61	10.50 ± 1.41	10.33 ± 1.71	0.11
Gender, % (number)							
Male	60% (9)	47% (17)	79% (11)	60% (9)	45% (10)	50% (9)	0.38
Female	40% (6)	53% (19)	21% (3)	40% (6)	55% (12)	50% (9)	–
SER at baseline (D)	− 2.83 ± 0.98	− 3.04 ± 0.89	− 2.98 ± 1.13	− 2.92 ± 0.91	− **2.68 ± 0.88**	− **2.65 ± 1.18**	0.68
SER at 2-year (D)	− 3.07 ± 0.83	− 3.44 ± 1.02	− 3.29 ± 1.15	− 3.94 ± 1.15	− **3.67 ± 0.95**	− **3.24 ± 1.33**	0.09
SER at 3-year (D)	− 3.19 ± 0.86	− 3.57 ± 1.08	− 3.73 ± 1.30	− 4.05 ± 1.34	− 3.78 ± 1.19	− 3.19 ± 1.47	0.25
SER at 6-year (D)	–	− 3.96 ± 1.42	− **4.28 ± 1.15**	–	− 3.92 ± 1.18	− **3.87 ± 1.53**	
AL at baseline (mm)	24.38 ± 0.90	24.68 ± 0.76	25.00 ± 0.80	24.59 ± 1.05	**24.62 ± 0.79**	**24.42 ± 0.86**	0.40
AL at 2-year (mm)	24.54 ± 0.83	24.90 ± 0.77	25.20 ± 0.72	25.16 ± 1.15	**25.21 ± 0.89**	**24.80 ± 0.86**	0.11
AL at 3-year (mm)	24.65 ± 0.84	25.00 ± 0.77	25.33 ± 0.76	25.28 ± 0.28	25.30 ± 0.95	24.83 ± 0.85	0.14
AL at 6-year (mm)	–	25.28 ± 0.81	**25.71 ± 0.69**	–	25.43 ± 1.01	**25.14 ± 0.87**	–

The bold figures in the rows of SER and AL represent the time of wearing SV spectacle lenses and the unbold figures represent the time of wearing DIMS spectacle lenses.

### Changes in SER and AL over 6 years of DIMS spectacle lens wear

**Figure 2:**
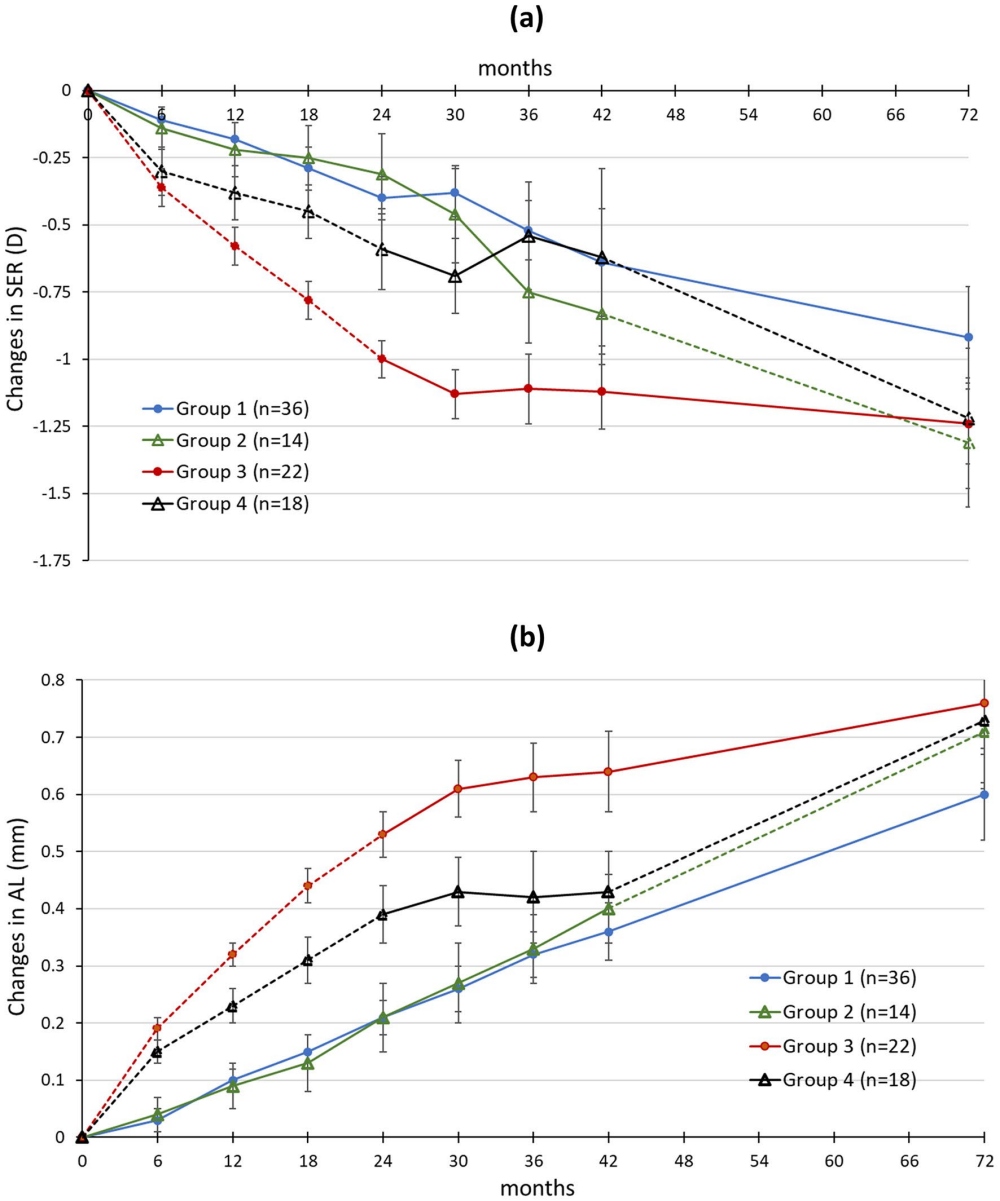
The mean and standard error of (**a**) myopia progression (changes in SER) and (**b**) axial elongation (changes in AL) from baseline to 6 years for Groups 1–4. The solid lines represent the time of wearing DIMS spectacle lenses and the dot lines represent the time of wearing single vision spectacle lenses.

Table 2 summarises the mean changes while eTable 3 summarises the cumulative changes in SER and AL from baseline to 6 years. The children in Group 1 (n = 36) showed the least myopia progression and axial elongation which were − 0.92 ± 1.15D (mean ± SD) and 0.60 ± 0.49 mm. Group 1 sustained a similar rate of myopia progression throughout 6 years (Fig. 2), and no statistically significant difference (p > 0.05) in myopia progression was found between the first 3 years and 4 to 6 years. Myopia progression in the first 3 years was − 0.52 ± 0.66D (annual rate: − 0.17D/year) while progression between 3 and 6 years was − 0.40 ± 0.71D (− 0.13D/year).

**Table 2:** Changes in the cycloplegic spherical equivalent refraction (SER) and axial length (AL) between different visits in Groups 1–4.

	DIMS		Control-to-DIMS	
	Group 1 (N = 36)	Group 2 (N = 14)	Group 3 (N = 22)	Group 4 (N = 18)
Time/SER (D) ± SD				
6-month	− 0.11 ± 0.31	− 0.14 ± 0.30	− **0.36 ± 0.31**	− **0.30 ± 0.38**
12-month	− 0.07 ± 0.29	− 0.08 ± 0.28	− **0.22 ± 0.28**	− **0.08 ± 0.28**
18-month	− 0.11 ± 0.36	− 0.03 ± 0.24	− **0.20 ± 0.25**	− **0.07 ± 0.31**
24-month	− 0.11 ± 0.31	− 0.05 ± 0.23	− **0.22 ± 0.22**	− **0.14 ± 0.36**
30-month	0.02 ± 0.28	− 0.15 ± 0.29	− 0.13 ± 0.31	− 0.10 ± 0.23
36-month	− 0.15 ± 0.39	− 0.29 ± 0.39	− 0.02 ± 0.36	0.16 ± 0.41
42-month	− 0.12 ± 0.42	− 0.08 ± 0.34	− 0.00 ± 0.27	− 0.07 ± 0.36
72-month	− 0.30 ± 0.65	− **0.48 ± 0.37**	− 0.13 ± 0.42	− **0.63 ± 0.49**
Time/AL(mm) ± SD				
6-month	0.03 ± 0.10	0.04 ± 0.12	**0.19 ± 0.08**	**0.15 ± 0.08**
12-month	0.07 ± 0.07	0.06 ± 0.07	**0.13 ± 0.07**	**0.08 ± 0.09**
18-month	0.04 ± 0.07	0.02 ± 0.11	**0.12 ± 0.07**	**0.08 ± 0.09**
24-month	0.07 ± 0.06	0.07 ± 0.07	**0.10 ± 0.06**	**0.08 ± 0.08**
30-month	0.05 ± 0.07	0.07 ± 0.07	0.08 ± 0.07	0.05 ± 0.05
36-month	0.05 ± 0.06	0.06 ± 0.06	0.02 ± 0.09	-0.02 ± 0.08
42-month	0.04 ± 0.07	0.07 ± 0.06	0.01 ± 0.10	0.01 ± 0.09
72-month	0.25 ± 0.24	**0.31 ± 0.21**	0.12 ± 0.13	**0.30 ± 0.19**

SER: spherical equivalent refraction, D = dioptres, AL: axial length. The bold figures represent the time of wearing SV spectacle lenses and the unbold figures represent the time of wearing DIMS spectacle lenses.

### Changes in SER and AL from 3.5 to 6 years

**Figure 3:**
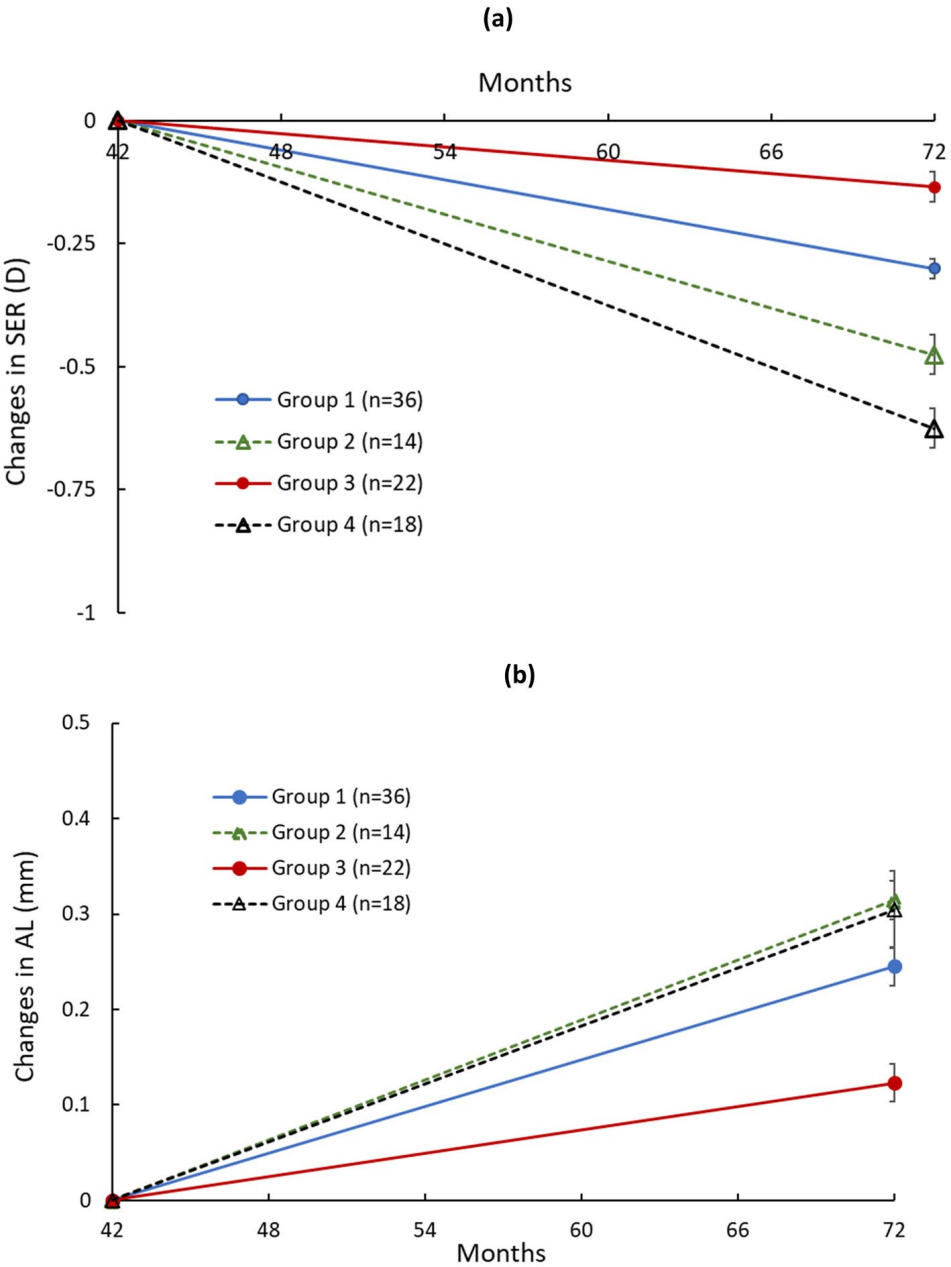
Changes in SER and AL between 3.5 to 6 years in Groups 1–4. The solid lines represent the time of wearing DIMS spectacle lenses and the dot lines represent the time of wearing single vision spectacle lenses.

Figure 3 shows the changes in SER and AL between 3.5 and 6 years in different groups. Group 3 showed the least myopia progression and axial elongation in the last 2.5 years among the groups. Both the DIMS lens groups (Group 1 and Group 3) had less myopia progression and axial elongation than the single vision lens groups (Group 2 and Group 4). Between the groups wearing DIMS lenses, Group 3 children showed slower myopia progression and axial elongation in the last 2.5 years than Group 1, but only the changes of AL (0.13 ± 0.46 mm, *p* = 0.023) showed statistically significant differences. Group 2 and Group 4 had similar changes in AL in the last 2.5 years. Although Group 4 exhibited faster myopia progression (Table 2) than Group 2, the differences were not statistically significant (*p* > 0.05).

The myopia progression and axial elongation between 3.5 and 6 years in Group 2 were − 0.48 ± 0.37D (− 0.19D/year) and 0.31 ± 0.21 mm (0.12 mm/year) and in Group 4 were − 0.63 ± 0.49D (-0.25D/year) and 0.30 ± 0.19 mm (0.12 mm/year). However, such an amount of myopia progression in Group 2 and Group 4 did not indicate a rebound effect. This can be observed that the duration of DIMS lens wear showed a flatter slope of myopia progression and axial elongation, and the slope of SV spectacle lens wear in the last 2.5 years did not show a faster progression rate when compared to those in the initial 2 years (Fig. 2). The treatment effect from DIMS spectacle lens was sustained.

### Myopia progression in individuals

In Group 1 (eFigure 1) 8 out of 36 children (22.2%) did not have any myopic progression (0 to + 0.50D) over 6 years; 3 of them started the DIMS spectacles wear at the age of 9 years, 2 at 10 years old, and 3 at 11 or 12 years old. Five children showed 0.25 to 0.50D of myopia reduction. Twelve children (33%) had an axial elongation of less than 0.3 mm over 6 years (~ 0.05 mm/year). A small portion of the children had low responses to the treatment, 8% had more than 3.00D of myopia progression (0.50D/year on average) and 11% had more than 1.2 mm of axial elongation over 6 years (0.20 mm/year).

### Age effect on slowing effect

Both myopia progression and axial elongation slowed with age for children in Group 1 (eFigure 2). eFigure 3 shows the changes in SER and AL over 6 years in different age groups, older children at enrolment gained better myopia control with DIMS lenses than younger children. And eTable 4 shows the mean changes in each age group.

### Visual functions after 6-year lens wear

Table 3 shows visual functions at the 6-year visit. There were no statistically significant differences in best-corrected VA, distance and near phoria, stereopsis, and AA (Kruskal–Wallis test, *p* > 0.05) among the 4 groups. All findings of visual function tests were within normal ranges. Long-term wear of DIMS spectacle lenses did not cause any adverse effects on the visual functions.

**Table 3:** Post-trial visual functions.

Mean ± SD	Group 1 (n = 36)	Group 2 (n = 14)	Group 3 (n = 22)	Group 4 (n = 18)	Kruskal–Wallis test, *P* value
BCVA (distance), Log MAR	− 0.09 ± 0.16	− 0.13 ± 0.26	− 0.12 ± 0.20	− 0.05 ± 0.09	0.53
Distance phoria, Δ	− 1.36 ± 2.00	− 0.86 ± 1.75	− 1.45 ± 1.82	− 0.67 ± 1.19	0.46
Near phoria, Δ	− 3.86 ± 5.00	− 3.86 ± 3.46	− 4.23 ± 5.02	− 2.72 ± 3.97	0.59
Stereoacuity, seconds of arc	25.14 ± 8.32	25.00 ± 7.84	24.32 ± 7.12	24.17 ± 7.52	0.94
Monocular amplitude of accommodation (right eye), D	17.06 ± 3.06	15.64 ± 2.79	16.49 ± 2.74	16.21 ± 2.88	0.33
Binocular AA, D	18.31 ± 2.44	17.71 ± 2.30	18.18 ± 2.79	17.87 ± 2.67	0.61

## Discussion

This study over 6 years (including the 2.5-year follow-up) on DIMS spectacle lens wear is one of the longest studies of myopia control intervention. The children who wore DIMS lenses over 6 years (Group 1) had − 0.92D of myopia progression (− 0.15D/year) and 0.60 mm of axial elongation (0.10 mm/year). There were no statistically significant differences in myopia progression during the first three years and the next three years. Although myopia progression slows with age, it was encouraging that the myopia control effect was still exhibited throughout the 6 years. On the other hand, the children (Group 2 and Group 4) who discontinued DIMS lens wear exhibited faster myopia progression and axial elongation compared to those who kept DIMS lens wear (Group 1 and Group 3). These findings support that the myopia control effect was sustained in the treatment groups. Both Group 1 and Group 3 were wearing DIMS spectacles for the last 2.5 years. The children in Group 3 wore the treatment lenses for 4 years (started after 2-year RCT) and started the treatment at an older age while those in Group 1 wore the DIMS lenses for 6 years. Surprisingly, Group 3 showed slower myopia progression and axial growth than Group 1.

A few RCTs of optical myopia interventions reported data over 3 years^13,18,19,21^. Cheng et al.^21^ found that Chinese-Canadian children who wore ordinary executive bifocals and prismatic bifocals showed − 1.25 ± 0.10D and − 1.01 ± 0.13D over 3 years, respectively, i.e., the myopia progressions were about 0.41D/ year and 0.34D/ year. The progression findings in the DIMS wearing children showed about 56 to 63% less than them. However, the children in that study had fast myopia progression before enrolment. A 5-year study indicated that orthokeratology could effectively retard axial elongation in children^13^. The elongation over 5 years was 0.99 ± 0.47 mm for the orthokeratology group (0.20 mm/year). For soft contact lenses, a 3-year study^19^ reported the absolute myopia progression and axial elongation in children wearing multifocal soft contact lenses were -0.60D and 0.39 mm (0.20D/year and 0.13 mm/year).

Only one clinical trial has been reported with data over 6 years^22^. The RCT of MiSight 1-day contact lenses showed myopia progression and axial elongation in the treatment group were − 0.51 ± 0.64D and 0.30 ± 0.27 mm (0.17D/year and 0.1 mm/year) over the first 3 years^11^. The children who completed the 3-year RCT were assigned to wear the treatment lenses for the other 3 years. The original control group changed to wear the same treatment lenses and exhibited a significant reduction in myopic progression from the previous SV 1-day contact lens wear. The results indicated that children who continued to wear dual-focus soft contact lenses showed myopia control effect sustained for up to 6 years. The prior treatment in the first 3 years did not affect later treatment efficacy (Table 4)^22^. Their mean myopia progression and axial elongation were − 0.92 ± 0.87D and 0.49 ± 0.39 mm, which were comparable to the findings from the children wearing the DIMS lenses continuously for 6 years (− 0.92 ± 1.15D and 0.60 ± 0.49 mm). Their study was carried out in multi-centres including children of different ethnicity whereas our study only included ethnic Chinese children. This might indicate that the race of the children might not be a factor to influence the efficacy of myopia control. Bullimore et al.^23^ evaluated the findings of different studies of myopia control and also suggested that no matter what is the race of the children, the benefit of any myopia control treatment seems to be the same.

**Table 4:** Comparison of SER and AL changes with a dual-focus contact lens.

Myopia control lenses	SER changes Mean ± SD (D)		AL changes Mean ± SD (mm)	
	first 3 years	3 to 6 years	first 3 years	3 to 6 years
Dual-focus contact lensy^22^	− 0.52 ± 0.64	− 0.45 ± 0.41	0.30 ± 0.28	0.22 ± 0.17
DIMS spectacle lens	− 0.52 ± 0.66	− 0.40 ± 0.72	0.32 ± 0.26	0.28 ± 0.28

Whilst treatments of myopia control are effective, there is a concern about a rebound effect which is an accelerated myopia progression or eye growth after discontinuing treatment as compared to the untreated children of similar ages, even to the point of counteracting the prior myopia control effect^24^. Most studies investigated the presence of any rebound effect after stopping the treatment from 6 to 12 months. This study is the first study to observe the myopia progression rate over 2.5 years after the discontinuation of myopia control. Our results revealed that the mean myopia progression and axial elongation were about 0.22D/year and 0.12 mm/year after stopping DIMS lens wear (combining Group 2 and Group 4), and such amount of myopia progression was clinically insignificant compared with children in the same age range^25–27^.

Thus, we conclude that there was no evidence of a rebound effect. Similarly, MiSight contact lenses^28^ and progressive additional lenses^29^ showed negligible myopic rebound after switching to SV contact lenses and SV spectacles for one year, respectively. Conversely, discontinuation of high-dose atropine (≥ 0.1%)^30^ led to more than 0.1 mm axial elongation than the control groups for one year, and discontinuation of orthokeratology lenses^31^ led to more than 0.07 mm axial elongation than using SV spectacle lenses over 6 months. The reason for the myopic rebound in these methods is unclear. On the other hand, the myopic defocus signal from optical devices, such as DIMS spectacle lenses and dual-focus contact lenses, seems to be relatively stable in resisting rebound.

Fan et al.^32^ reported the mean rate of myopia progression in Hong Kong children aged 5 to 16 years was − 0.63 D/year. Sankaridurg et al.^26^ and Donovan et al.^27^ reviewed the previous epidemiological studies on myopia prevalence extensively and constructed an equation for estimating the annual rate of myopia progression in Asian children (eTable 5). Both studies indicated that the younger the age the greater the myopia progression. Donovan’s nonsensical quadratic model predicted that myopia progression does not slow but continues to accelerate at the age of 15 years^27^. In our study, all age subgroups in Group 1 showed a slower annual rate of myopia progression than the rate in those studies. A study on European children estimated the annual myopia progression at − 0.50 D for the age of fewer than 10 years and − 0.38 D for the age of 10 to 12 years^33^. The mean annual myopia progression in the purely DIMS group (Group 1: − 0.15D) was much less than the general populations of children with similar ethnicity and Europeans at similar ages.

Similar to the first 3 years^15,20^, the 6-year results of Group 1 also indicated that the older children seemed to exhibit a better treatment effect with the DIMS spectacle lens wear. Children with a baseline age of 10 to 13 years had almost no myopia progression and less than 0.08 mm/year of axial elongation at 42 to 72 months (eFigure 3) and this amount of axial elongation has been suggested to be the physiological eye growth^34,35^. Age has been documented as an associated factor with myopia progression^25^. Thus myopia control using DIMS spectacle lenses could slow the faster myopia progression in earlier childhood and then maintain almost no myopia progression in the latter stages of their childhood.

A recent review of myopia control studies indicated that age is not a factor affecting the efficacy of myopia control modality^36^. However, our study found that the older children progressed slower than the younger ones. eFigure 3 shows the myopia progression of DIMS lens wearers of each age group from baseline to 6 years. The 8-year-old group always showed more myopia progression and axial elongation. In the 42 to 72 months, the myopic progression in the 8-year-old group was still faster than in other age groups. We have discussed this observation in two publications^37,38^ that relative peripheral refraction (RPR) at the start of the treatment has some impact on myopia control outcomes. Most children with younger enrolment age (aged 8 years) in the 2-year RCT of DIMS lenses had myopic RPR at baseline^37,38^. They showed less myopia control effects compared with the other age groups who had baseline hyperopic RPR and this impact continues to older age despite continuation with treatment lenses. A possible explanation for the variation in myopia control effectiveness between ages could be due to the interaction of the RPR profile and the imposed myopia defocus during treatment. The myopia control effect depends on the counterbalance of the hyperopic defocus from the eye by the myopic defocus from the DIMS lens. The ideal situation is to shift the eye’s hyperopic defocus to become myopic defocus. Myopic children with baseline myopic RPR when combined with the myopic defocus of + 3.5D from the treatment lens may be receiving too much myopic defocus at the mid-periphery retina, and this situation may result in an overall more blur peripheral image and such blur could be beyond the threshold of signal detection, and myopia control would therefore be less effective in this age group^37,38^. In the same way, Group 3 and Group 4 started to wear DIMS lenses at 24 months, these two groups showed better myopia control than Group 1. It could also be related to the RPR profile of Groups 3 and 4, having more hyperopic RPR than Group 1 who were shown to have more myopic RPR at the start of the treatment (eTable 6a,b,c). From these findings, we expect that there could be a cap on the amount of imposed myopic defocus that could slow eye growth and myopia progression. And this is uncommon in animal myopia research^37,38^. Also, the children in the older age groups became older than 16 years during that period, and their myopia may have become more stable as per normal eye development.

There were some limitations in this study. First, the children self-selected their choices of spectacle lens wear in the last 2.5 years, so there were four separate groups with different spectacles-wearing combinations during the 6 years. Therefore, the study was not randomized and had selection bias. However, it did benefit from the comparison of the myopia progression trend in the treatment groups with the groups stopping the DIMS lens wear in the last 2.5 years. Second, due to various reasons, it was not possible to continue with every 6-month monitoring and the study was interrupted until three and a half years after the onset of the study. In addition, the sample size in each group became smaller, and the attrition rate (25%) was relatively high due to the long follow-up period and the unexpected COVID pandemic which has caused reluctance for many participants to re-join the research. However, we believe it is a minor limitation as the comparison between the dropouts and those who completed the 6-year study showed no substantial differences (Table 1).

In addition, Group 3 and Group 4 showed differences in the rate of myopia progression (Fig. 2) in the first 2 years. Both groups were wearing SV spectacles in the 2-year RCT and they were expected to have a similar trend in SER and AL changes. There might be other subtle and unmeasured differences between these two groups, so such differences limit the comparison and interpretation of subsequent effect in the last 2.5 years. When considering the rebound effect, we extrapolated the progression trend from the original control group in the 2 years RCT and found that when children stopped the DIMS lens wear and revert to single vision lens wear, there was no sharp change in the rate of myopia progression (comparing the slopes of the first 2 years RCT control with the last 2.5 years of single vision lens wear). In assessing the rebound effect, data from the natural myopia progression for people who are uncontrolled, ideally matched for age and SER should be used for comparison. However, the current participants in all four groups have received certain degrees of intervention so their data in years 3.5 to 6 could not be treated as control. With only preliminary, single-group data, while there is a suggestion that there is no rebounding effect, further studies with bigger sample sizes are needed to confirm this observation.

## Conclusions

Our study demonstrated that DIMS spectacle lenses provided a sustained effect of slowing myopia progression and axial elongation in myopic children who wore DIMS lenses for up to 6 years. On the other hand, children who discontinued the treatment did not show evidence of a rebound effect. The findings of visual functions indicated that long-term wear of DIMS spectacle lenses did not show any adverse effect, we conclude that DIMS spectacle lenses are safe to be used as a clinical intervention for childhood myopia control.

## Supplementary Information

Supplementary Information.

## Data Availability

The datasets used and/or analysed during the current study are available from the corresponding author on reasonable request.
