# Supplementary material for: Long-term myopia control effect and safety in children wearing DIMS spectacle lenses for 6 years
Source: Sci Rep. 2023 Apr 4;13:5475. doi: 10.1038/s41598-023-32700-7 (PMC10073092; doi:10.1038/s41598-023-32700-7)
Supplement: Supplementary file 1 — Supplementary Information. [file 41598_2023_32700_MOESM1_ESM.docx]

**Supplementary information**

**Title: Long-term myopia control effect and safety in children wearing DIMS spectacle lenses for 6 years**

**Authors**: Carly Siu Yin Lam, Wing Chun Tang, Han Yu Zhang, Paul H Lee, Dennis Yan Yin Tse, Hua Q, Natalia Vlasak & Chi Ho To

**Supplementary Results**

**eTable 1**. Groups according to types of lens wear over the 6 years.

**eTable 2**. The number of dropouts and children who completed the 6-year follow-up.

**eTable 3.** Cumulative changes in the cycloplegic spherical equivalent refraction (SER) and axial length (AL) from baseline to 72 months for Groups 1-4.

**eTable 4.** Mean changes in spherical equivalent refraction (SER) and axial length (AL) over 6 years for different age groups.

**eTable 5.** Mean annual myopia progression rate in different age groups of different studies.

**eTable 6a.** Comparison of RPR at 10N at 24 months among Groups 1-4 after adjusting for baseline age, gender, baseline RPR at 10N, and myopia progression over the first 2 years.

**eTable 6b.** Comparison of RPR at 20N at 24 months among Groups 1-4 after adjusting for baseline age, gender, baseline RPR at 20N, and myopia progression over the first 2 years.

**eTable 6c.** Comparison of RPR at 30N at 24 months among Groups 1-4 after adjusting for baseline age, gender, baseline RPR at 30N, and myopia progression over the first 2 years.

**eFigure 1.** Distributions of myopia progression and axial elongation in the children wearing DIMS spectacles for 6 years (Group 1, n =36).

**eFigure 2.** Changes in SER and AL with the age of enrolment in Group 1 children who wore DIMS spectacle lenses for 6 years.

**eFigure 3.** Mean changes in SER and AL over 6 years for different ages in Group 1 (wore DIMS spectacle lenses for 6 years).

**eTable 1**. Groups according to types of lens wear over the 6 years.

|  | **Type of spectacle lens wear** | | | | | | |
| --- | --- | --- | --- | --- | --- | --- | --- |
|  | **Year 1** | **Year 2** | **Year3** | **Year 3.5** | **Year 4** | **Year 5** | **Year 6** |
| **Group 1** | DIMS spectacle lens (6 years) | | | | | | |
| **Group 2** | DIMS spectacle lens (3.5 years) | | | | SV spectacle lens (2.5 years) | | |
| **Group 3** | SV spectacle lens  (2 years) | | DIMS spectacle lens (4 years) | | | | |
| **Group 4** | SV spectacle lens  (2 years) | | DIMS spectacle lens  (1 year) | | SV spectacle lens (2.5 years) | | |

**eTable 2.** The number of dropouts and children who completed the 6-year follow-up.

| **Number of subjects** | **DIMS**  **(Groups 1 and 2)** | **Control-to-DIMS**  **(Groups 3 and 4)** | **Total** |
| --- | --- | --- | --- |
| Invited after the 3^rd^ year of study | 65 | 55 | 120 |
| Dropouts: Not joined or excluded from this study | 14 | 14 | 28 |
| Missed data | 1 | 1 | 2 |
| Completed 6-year follow-up | 50 | 40 | 90 |

**eTable 3.** Cumulative changes in the cycloplegic spherical equivalent refraction (SER) and axial length (AL) from baseline to 72 months for Groups 1-4.

|  | **DIMS** | | **Control-to-DIMS** | |
| --- | --- | --- | --- | --- |
| Time/ SER (D) ± SD | **Group 1**  **(N= 36)** | **Group 2**  **(N=14)** | **Group 3**  **(N=22)** | **Group 4**  **(N=18)** |
| 6-month | -0.11 ± 0.31 | -0.14 ± 0.30 | **-0.36 ± 0.31** | **-0.30 ± 0.38** |
| 12-month | -0.18 ± 0.34 | -0.22± 0.38 | **-0.58 ± 0.31** | **-0.38 ± 0.43** |
| 18-month | -0.29 ± 0.48 | -0.25 ± 0.48 | **-0.78 ± 0.33** | **-0.45 ± 0.44** |
| 24-month | -0.40 ± 0.47 | -0.31 ± 0.57 | **-1.00 ± 0.32** | **-0.59 ± 0.63** |
| 30-month | -0.38 ± 0.54 | -0.46 ± 0.67 | -1.13 ± 0.44 | -0.69 ± 0.60 |
| 36-month | -0.52 ± 0.66 | -0.75 ± 0.70 | -1.11± 0.62 | -0.54 ± 0.85 |
| 42-month | -0.64 ± 0.73 | -0.83 ± 0.70 | -1.11± 0.63 | -0.60 ± 0.93 |
| 72-month | -0.92 ± 1.15 | -**1.31 ± 0.90** | -1.24 ± 0.71 | **-1.22 ± 1.10** |
| Time/ AL(mm) ± SD | **Group 1**  **(N= 36)** | **Group 2**  **(N=14)** | **Group 3**  **(N=22)** | **Group 4**  **(N=18)** |
| 6-month | 0.03 ± 0.10 | 0.04 ± 0.12 | **0.19 ± 0.08** | **0.15 ± 0.08** |
| 12-month | 0.10 ± 0.14 | 0.09 ± 0.16 | **0.32 ± 0.10** | **0.23 ± 0.15** |
| 18-month | 0.15 ± 0.17 | 0.13 ± 0.19 | **0.44 ± 0.14** | **0.31 ± 0.18** |
| 24-month | 0.21 ± 0.20 | 0.21 ± 0.23 | **0.53 ± 0.18** | **0.39 ± 0.22** |
| 30-month | 0.26 ± 0.24 | 0.27 ± 0.25 | 0.61 ± 0.23 | 0.43 ± 0.25 |
| 36-month | 0.32 ± 0.26 | 0.33 ± 0.24 | 0.63 ± 0.28 | 0.42 ± 0.29 |
| 42-month | 0.36 ± 0.29 | 0.40 ± 0.24 | 0.64 ± 0.34 | 0.43 ± 0.36 |
| 72-month | 0.60 ± 0.49 | **0.71 ± 0.37** | 0.76 ± 0.43 | **0.73 ± 0.49** |

SER: spherical equivalent refraction, D = dioptres, AL: axial length. The bold figures represent the time of wearing SV spectacle lenses and the unbold figures represent the time of wearing DIMS spectacle lenses.

**eTable 4.** Mean changes in spherical equivalent refraction (SER) and axial length (AL) over 6 years for different age groups.

| **Enrolment age (years)** | **Changes in SER over 6 years (D)** | **Changes in AL over 6 years (mm)** |
| --- | --- | --- |
| 8 (n= 7) | -1.90 ± 1.52 | 1.08 ± 0.48 |
| 9 (n=12) | -1.05 ± 1.18 | 0.70 ± 0.38 |
| 10 (n= 7) | -0.42 ± 0.55 | 0.40 ± 0.17 |
| 11 (n= 4) | -0.75 ± 0.96 | 0.46 ± 0.25 |
| 12 (n= 5) | -0.19 ± 0.35 | 0.20 ± 0.07 |
| 13 (n= 1) | -0.31 ± 0.00 | 0.05 ± 0.00 |

**eTable 5**. Mean annual myopia progression rate in different age groups of different studies.

|  | **Myopia progression per year (D): Mean ± SD** | | | |
| --- | --- | --- | --- | --- |
| **Age (years)** | **Control in first**  **2-year (n=81)^15^** | **Sankaridurg’s estimation^25^** | **Donovan’s estimation^26^** |  |
| 8 | -0.67 ± 0.29 (n=12) | -0.81 | -0.94 |  |
| 9 | -0.56 ± 0.17 (n=20) | -0.71 | -0.78 |  |
| 10 | -0.52 ± 0.27 (n=22) | -0.62 | -0.66 |  |
| 11 | -0.41 ± 0.22 (n=11) | -0.53 | -0.56 |  |
| 12 | -0.47 ± 0.18 (n=11) | -0.46 | -0.50 |  |
| 13 | -0.44 ± 0.50 (n=5) | -0.38 | -0.46 |  |
| 14 | - | -0.32 | -0.44 |  |
| 15 | - | -0.26 | -0.46 |  |

p-value <0.05 was considered to be statistically significant.

**eTable 6a.** Comparison of RPR at 10N at 24 months among Groups 1-4 after adjusting for baseline age, gender, baseline RPR at 10N, and myopia progression over the first 2 years.

| (I) Group | (J) Group | Mean Difference (I-J) | SE  ( standard error) | p-value | 95% Confidence Interval for Difference | |
| --- | --- | --- | --- | --- | --- | --- |
|  |  |  |  |  | Lower Bound | Upper Bound |
| 1 | 2 | 0.02 | 0.18 | 1.00 | -0.45 | 0.50 |
|  | 3 | -0.68 | 0.17 | 0.00 | -1.14 | -0.23 |
|  | 4 | -0.51 | 0.16 | 0.02 | -0.95 | -0.06 |
| 2 | 1 | -0.02 | 0.18 | 1.00 | -0.50 | 0.45 |
|  | 3 | -0.71 | 0.21 | 0.01 | -1.28 | -0.13 |
|  | 4 | -0.53 | 0.20 | 0.07 | -1.08 | 0.02 |
| 3 | 1 | 0.68 | 0.17 | 0.00 | 0.23 | 1.14 |
|  | 2 | 0.71 | 0.21 | 0.01 | 0.13 | 1.28 |
|  | 4 | 0.18 | 0.18 | 1.00 | -0.31 | 0.66 |
| 4 | 1 | 0.51 | 0.16 | 0.02 | 0.06 | 0.95 |
|  | 2 | 0.53 | 0.20 | 0.07 | -0.02 | 1.08 |
|  | 3 | -0.18 | 0.18 | 1.00 | -0.66 | 0.31 |

p-value <0.05 was considered to be statistically significant.

**eTable 6b.** Comparison of RPR at 20N at 24 months among Groups 1-4 after adjusting for baseline age, gender, baseline RPR at 20N, and myopia progression over the first 2 years.

| (I) Group | (J) Group | Mean Difference (I-J) | SE  ( standard error) | p-value | 95% Confidence Interval for Difference | |
| --- | --- | --- | --- | --- | --- | --- |
|  |  |  |  |  | Lower Bound | Upper Bound |
| 1 | 2 | 0.08 | 0.24 | 1.00 | -0.57 | 0.73 |
|  | 3 | -0.81 | 0.23 | 0.01 | -1.44 | -0.18 |
|  | 4 | -0.63 | 0.22 | 0.03 | -1.23 | -0.03 |
| 2 | 1 | -0.08 | 0.24 | 1.00 | -0.73 | 0.57 |
|  | 3 | -0.89 | 0.29 | 0.02 | -1.66 | -0.11 |
|  | 4 | -0.71 | 0.27 | 0.07 | -1.45 | 0.03 |
| 3 | 1 | 0.81 | 0.23 | 0.01 | 0.18 | 1.44 |
|  | 2 | 0.89 | 0.29 | 0.02 | 0.11 | 1.66 |
|  | 4 | 0.18 | 0.24 | 1.00 | -0.48 | 0.84 |
| 4 | 1 | 0.63 | 0.22 | 0.03 | 0.03 | 1.23 |
|  | 2 | 0.71 | 0.27 | 0.07 | -0.03 | 1.45 |
|  | 3 | -0.18 | 0.24 | 1.00 | -0.84 | 0.48 |

p-value <0.05 was considered to be statistically significant.

**eTable 6c.** Comparison of RPR at 30N at 24 months among Groups 1-4 after adjusting for baseline age, gender, baseline RPR at 30N, and myopia progression over the first 2 years.

| (I) Group | (J) Group | Mean Difference (I-J) | SE  ( standard error) | p-value | 95% Confidence Interval for Difference | |
| --- | --- | --- | --- | --- | --- | --- |
|  |  |  |  |  | Lower Bound | Upper Bound |
| 1 | 2 | -0.11 | 0.32 | 1.00 | -0.96 | 0.75 |
|  | 3 | -0.99 | 0.31 | 0.01 | -1.82 | -0.17 |
|  | 4 | -0.85 | 0.30 | 0.03 | -1.66 | -0.04 |
| 2 | 1 | 0.11 | 0.32 | 1.00 | -0.75 | 0.96 |
|  | 3 | -0.89 | 0.38 | 0.13 | -1.91 | 0.14 |
|  | 4 | -0.74 | 0.37 | 0.30 | -1.75 | 0.27 |
| 3 | 1 | 0.99 | 0.31 | 0.01 | 0.17 | 1.82 |
|  | 2 | 0.89 | 0.38 | 0.13 | -0.14 | 1.91 |
|  | 4 | 0.15 | 0.32 | 1.00 | -0.73 | 1.02 |
| 4 | 1 | 0.85 | 0.30 | 0.03 | 0.04 | 1.66 |
|  | 2 | 0.74 | 0.37 | 0.30 | -0.27 | 1.75 |
|  | 3 | -0.15 | 0.32 | 1.00 | -1.02 | 0.73 |

p-value <0.05 was considered to be statistically significant.


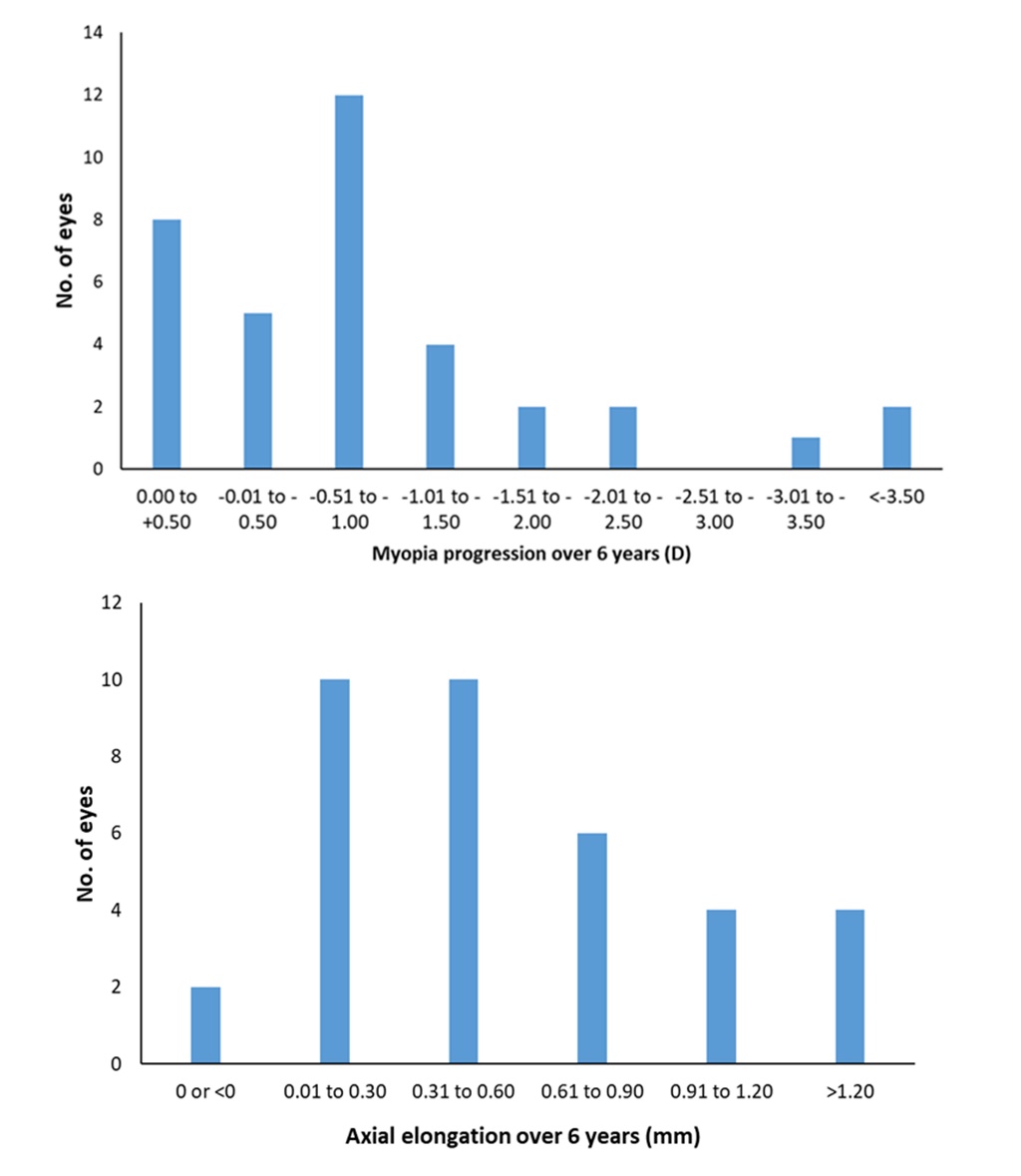


**eFigure 1**. Distributions of myopia progression and axial elongation in the children wearing DIMS spectacles for 6 years (Group 1, n =36).


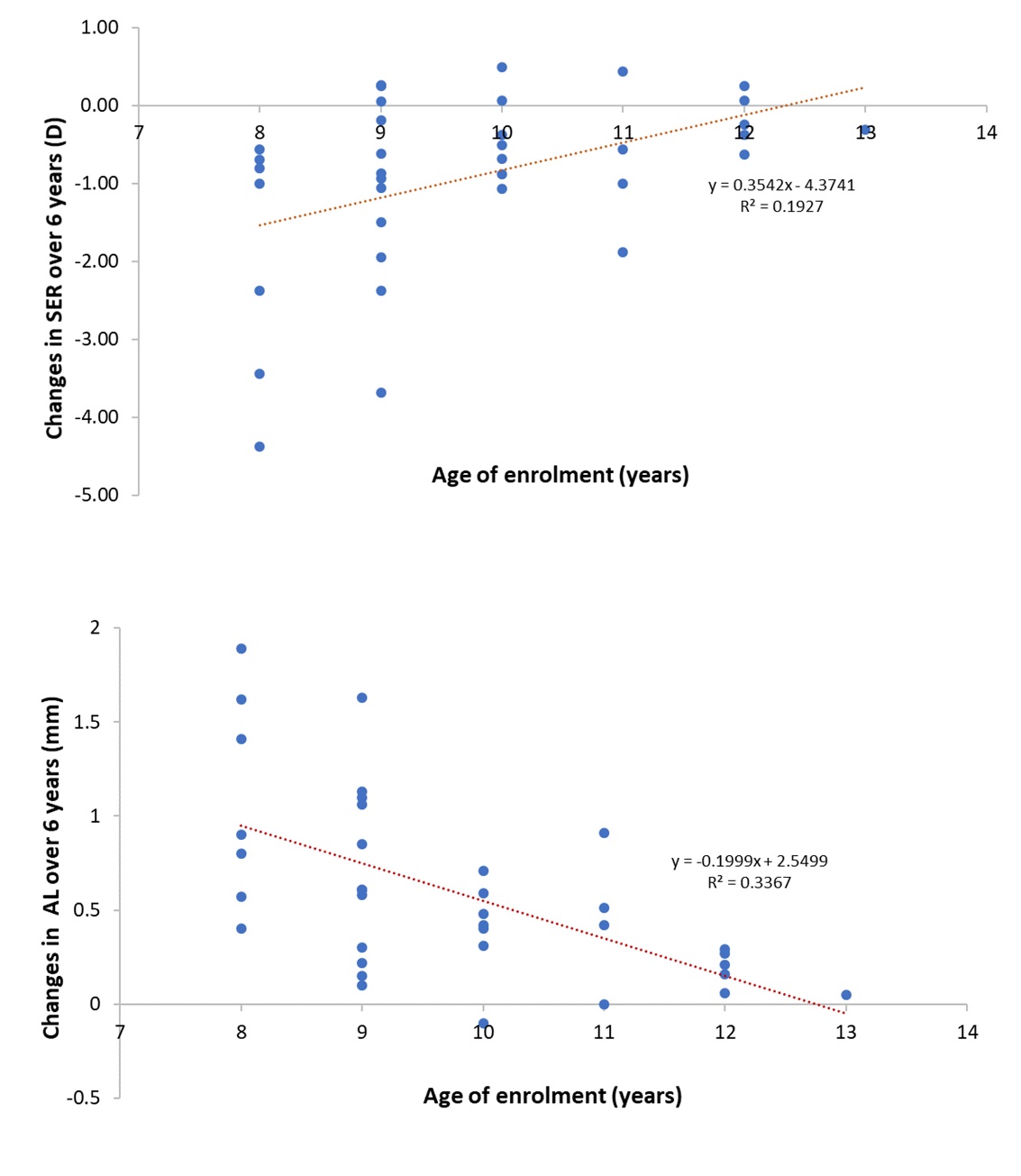


**eFigure 2.** Changes in SER and AL with the age of enrolment in Group 1 children who wore DIMS spectacle lenses for 6 years.


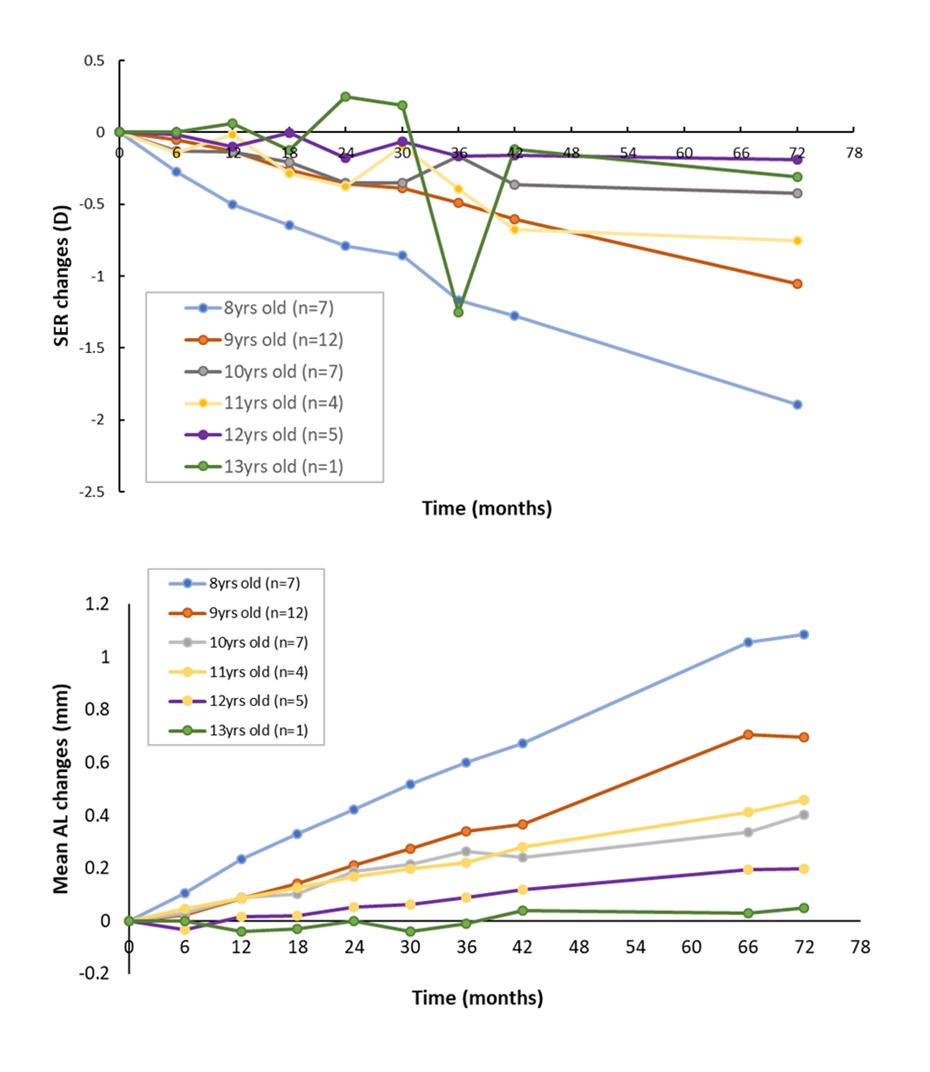


**eFigure 3.** Mean changes in SER and AL over 6 years for different ages in Group 1 (wore DIMS spectacle lenses for 6 years).
